# In Vitro Silencing of MHC-I in Keratinocytes by Herpesvirus US11 Protein to Model Alloreactive Suppression

**DOI:** 10.3390/ebj6030047

**Published:** 2025-08-21

**Authors:** Frederik Schlottmann, Sarah Strauß, Peter Maria Vogt, Vesna Bucan

**Affiliations:** Department of Plastic, Aesthetic, Hand and Reconstructive Surgery, Hannover Medical School, Carl-Neuberg-Strasse 1, 30625 Hanover, Germany

**Keywords:** allografts, burns, immunology, recombinant proteins, skin transplantation

## Abstract

Background: Secondary rejection remains a major obstacle in skin allografting. Some viruses, such as human herpesvirus and cytomegalovirus, evade immune detection through proteins like the unique short glycoprotein 11 (US11), which down-regulates major histocompatibility complex (MHC) class I expression. This study explores the use of recombinant US11 protein as a biopharmaceutical approach to reduce MHC-I expression and thus decrease alloreactivity in human primary keratinocytes. Methods: Human keratinocytes were treated with recombinant US11 protein, and MHC-I expression was assessed via Western blot and flow cytometry. To evaluate immunomodulatory effects, US11-stimulated keratinocytes were co-cultured with peripheral blood mononuclear cells (PBMCs), and interferon-gamma (IFN-γ) levels were measured by ELISA. Additionally, ex vivo human skin tissue was stimulated with US11 to assess long-term MHC-I modulation. Results: US11 treatment significantly reduced MHC-I surface expression in keratinocytes. Co-cultures showed decreased IFN-γ secretion, indicating lower T cell activation. Human skin tissue stimulated with US11 exhibited reduced MHC-I expression after 7 days. Conclusions: This proof-of-concept study suggests that recombinant US11 protein may serve as an effective biopharmaceutical to reduce keratinocyte immunogenicity. Further in vitro and in vivo studies are warranted to validate its potential for clinical application in skin transplantation.

## 1. Introduction

Burns and their complex therapy are among the most prevalent and devastating injuries, especially in developing countries, often resulting in significant morbidity [1]. Surgical burn wound therapy typically involves wound debridement to establish aseptic conditions, followed by defect coverage [2]. The spectrum of defect coverage ranges from the use of commercially available skin substitutes to free, microsurgical flap plasties [3]. Autologous split-thickness skin grafting has proven to be a safe, low-risk, and well-established method of defect coverage in burns [4,5]. When there are not enough donor areas available for autologous skin transplantation in the case of extensive burn wounds, several alternative reconstruction procedures can be used. These alternatives include xenogeneic and allogeneic skin grafting for temporary defect coverage [4]. Allogeneic skin grafts serve as a temporary wound covering for extensive burns when adequate autologous donor sites are unavailable. They effectively protect the wound from infection and prepare the wound bed for subsequent autologous grafting [6]. However, their application is challenged by the risk of immunological rejection, infection, and increased scarring [7]. Given that severely burned patients are typically immunocompromised in the acute phase, allografts often achieve temporary integration but are subsequently rejected upon immune system reconstitution [8]. Achieving reliable clinical tolerance for permanent skin allografts has been elusive [9]. T cell-mediated mechanisms, along with B cell and natural killer (NK) cell activation, play significant roles in the rejection of allografts [10].

Major histocompatibility complex (MHC) molecules are critical in antigen and antibody interactions. MHC-I molecules, expressed on all nucleated cells, sample antigen peptides and signal the cell’s state to immune effector cells like CD8+ T cells, T lymphocytes, and NK cells [10,11,12]. Due to the complex genetic polymorphism of the MHC encoding human leukocyte antigen (HLA) genes, a whole genomic knockout has not been feasible [12,13]. Advances in tissue engineering and transplant immunology have led to promising therapeutic approaches [14,15]. Strategies such as creating HLA homozygous cell banks or generating histocompatible, patient-specific pluripotent stem cells aim to overcome immunological issues in allotransplantations [13,16]. While MHC compatibility between recipients and donors has significantly improved organ transplants, a perfect graft–donor match is still unachievable due to genomic diversity and donor organ supply–demand mismatches [17,18,19,20]. The necessary level of MHC-I expression reduction to evade host immune response is uncertain. Reports suggest that as few as 1 to 200 MHC-I molecules per cell can activate CD8+ cytotoxic T cells, whereas complete or near-complete elimination of MHC-I can induce NK cell-mediated apoptosis [21,22,23,24]. Consequently, most allotransplantations such as solid organs require immunosuppressive drugs like calcineurin inhibitors, mechanistic targets of rapamycin inhibitors, and biologicals to manage alloreactivity [25,26].

Various plasmids and viral vectors have been investigated to influence keratinocyte alloreactivity, aiming to create off-the-shelf donor cells invisible to the host immune system [22,27,28]. Strategies include introducing genes encoding key immunomodulatory molecules such as MHC-I [29]. For example, β2-microglobulin (β2m)-deficient mice showed a reduced rejection of allogeneic skin grafts, pancreatic islets, and heart and liver transplants [30,31,32,33]. Viral vectors and plasmids used for keratinocyte transfection also showed reduced alloreactivity and prolonged allograft survival [15,22,34].

Viruses like human cytomegalovirus (HCMV) and human herpes virus (HHV) have developed strategies to reduce MHC-I expression on infected cells, evading host immune responses [35,36]. HCMV’s and HHV’s MHC-I down-regulating genes, particularly unique short glycoprotein (US) 2, US3, US6, and US11, have been studied extensively [35,37,38]. US2 and US11 redirect MHC-I heavy chains for degradation by proteasomes, reducing CD8+ T cell activation but still allowing NK cell recognition [38,39,40]. Mice infected with HCMV virus vaccines containing US2 and US11 showed reduced CD8+ cytotoxic T cell activation as an indicator for host cellular immune response, but the reduction in CD8+ cytotoxic T cell activation ranged between 25 to 50% [41]. US gene expression and thereby reduced MHC- I expression levels were shown to be sufficient, as modified cells were still recognized by NK cells [42,43].

Transfecting human primary keratinocytes with US11 HCMV viral vectors transiently reduced MHC-I expression, potentially useful for creating allogeneic skin substitutes [15]. US proteins represent a promising approach to reducing MHC-I expression and allograft rejection. Gene therapeutic approaches using viral vectors offer quick proof-of-concept with low financial expenditure, but biopharmaceuticals are safer and easier to work with according to laws and regulations [44]. Recombinant biopharmaceuticals can avoid the negative effects of viral vectors, such as RNA interference and inheritance to subsequent generations [45,46]. There are no studies in the literature to date that have investigated the topically applied US11 protein as a biopharmaceutical for reducing MHC-I expression. Using recombinant synthesized US proteins to reduce MHC-I expression could effectively decrease alloreactivity in allogeneic keratinocytes. Over 400 recombinant protein-based products are approved as biopharmaceuticals, including six top-selling antibodies or antibody-derived proteins globally [47]. Generating universal cells that evade host immune responses using recombinant US11 proteins without immunosuppressive drugs would be a significant milestone in transplant immunology, offering a wide range of clinical applications.

## 2. Materials and Methods

### 2.1. Cell Culture

Human primary dermal keratinocytes (provitro, Berlin, Germany) were cultured following standardized internal protocols in serum-free keratinocyte growth medium (provitro, Berlin, Germany), supplemented with manufacturer-provided supplements (provitro, Berlin, Germany) and 10% fetal bovine serum. Keratinocytes were incubated at 37 °C in a humidified atmosphere with 5% CO_2_. The culture medium was changed thrice per week. Cells were subcultured at a ratio of 1:3 when they reached 70–80% confluence using Dispase II (provitro, Berlin, Germany). The experiments were conducted with keratinocytes at passage 3.

### 2.2. Recombinant US11 Proteins and Stimulation Procedure

Recombinant US11 proteins from human herpes virus 1 (Cusabio, Huston, TX, USA) were used, with the amino acid sequence from the Protein Database (GenBank: AAV88367.1). The three-dimensional protein structure was simulated using the SWISS-MODEL server (SWISS-MODEL, Basel, Switzerland) based on standardized protocols [48,49]. Keratinocytes were co-cultured with 0.1 mg/mL recombinant US11 proteins. Experiments were performed in triplicate.

### 2.3. Western Blotting

Total protein was extracted from keratinocytes stimulated with recombinant US11 proteins after 2, 4, and 6 h. Non-stimulated keratinocytes served as controls. Protein extraction used radioimmunoprecipitation assay buffer with additional reagents according to standardized protocols. Protein samples (25 µg each) were separated on 15% SDS-PAGE and transferred to PVDF membranes (Merck Millipore, Darmstadt, Germany). Immunoblotting used monoclonal rabbit anti-MHC class I + HLA-A + HLA-B (abcam, Cambridge, UK) and monoclonal mouse anti-alpha tubulin (Sigma Aldrich, Darmstadt, Germany) antibodies. Secondary antibodies were Odyssey 680/800 nm conjugates (Li-Cor BioSciences, Lincoln, NE, USA). Signals were visualized using the Odyssey Infra-Red Imaging System (Li-Cor BioSciences, Lincoln, NE, USA). All experiments were performed in triplicate to obtain significant results.

### 2.4. IFN-γ Enzyme-Linked Immunosorbent Assay (ELISA)

To assess the immunogenic potential of stimulated keratinocytes, an IFN-γ assay was performed with peripheral blood mononuclear cells (PBMCs) (ATTC, Manassas, VA, USA). PBMCs were cultured in RPMI 1640 medium (Gibco, ThermoFisher Scientific, Darmstadt, Germany) with additives specified in the manufacturer’s datasheet for PBMCs. Keratinocytes were stimulated with recombinant US11 proteins as described above (0.1 mg/mL) and afterwards with PBMCs for 24 h. The IFN-γ ELISA (Invitrogen, Eugene, OR, USA) was conducted according to the manufacturer’s instructions. Fluorescence was measured at 485 nm (excitation) and 530 nm (emission) using a Tecan GENios plate reader (Tecan Schweiz, Zuerich, Switzerland). Three samples from each study group were randomly selected for measurement (first, second, and third run). Each sample was measured three times. Results were analyzed using Microsoft^®^ Excel for Mac 2021 software (Version 16.86) (Microsoft Corporation, Redmond, WA, USA).

### 2.5. Flow Cytometry

MHC-I expression levels were measured using flow cytometry after 2 and 6 h of stimulation with US11 proteins. Non-stimulated cells were controls. Cells were detached using Dispase II (provitro, Berlin, Germany), washed with PBS, and blocked with 1% bovine serum albumin (Sigma Aldrich, Darmstadt, Germany). A total of 5000 cells per sample were selected for flow cytometry. Cells were labeled with primary antibodies (monoclonal rabbit anti-MHC class I + HLA-A + HLA-B antibody (abcam, Cambridge, UK)) for 50 min at 4 °C with a final dilution of 1:10. After incubation, cells were washed with PBS following incubation with fluorochrome-labeled secondary antibodies (Santa Cruz, Heidelberg, Germany) for 45 min at 4 °C with a final dilution of 1:5. Analysis was performed with a FC500 flow cytometer (Beckman Coulter, Krefeld, Germany). All experiments were performed in triplicate.

### 2.6. Skin Tissue Sampling and Immunofluorescence

The skin tissue was obtained as an allogeneic donation from abdominoplasty procedures performed at Hannover Medical School’s Department of Plastic, Aesthetic, Hand and Reconstructive Surgery, with all donors providing written consent. The experiments were ethically approved and conducted in accordance with the Declaration of Helsinki (ethics committee approval number: 3475-2017, dated 2 March 2017). Human skin tissue of three donors was cultured and treated with recombinant US11 proteins (0.1 mg/mL) every 2 or 6 h for 7 days. Tissue was fixed in 4% buffered formalin (Carl Roth, Karlsruhe, Germany) and 10 µm longitudinal cryosections were cut and mounted on Silane Prep glass slides (Sigma, St. Louis, MO, USA). Immunostaining was performed with monoclonal rabbit anti-MHC class I + HLA-A + HLA-B antibodies (abcam, Cambridge, UK; final diluation 1:100), followed by goat anti-rabbit IgG-Alexa Fluor 488 (Invitrogen, Eugene, OR, USA; dilution 1:1000) secondary antibody. Sections were coverslipped with DAPI-containing mounting medium (VectaShield, Vector Laboratories, Burlingame, CA, USA) and analyzed with a Zeiss Axiovert 200 M microscope (Carl Zeiss, Oberkochen, Germany) and associated software.

## 3. Results

### 3.1. Recombinant US11 Proteins and Potential Three-Dimensional Protein Structure

**Figure 1:**
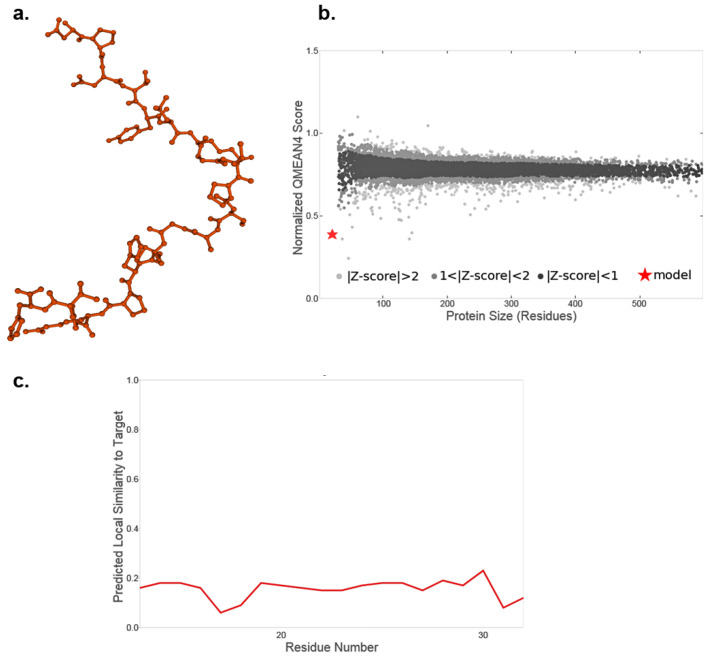
Protein structure of recombinant US11 protein. The potential three-dimensional protein structure of the recombinant US11 protein was simulated using the SWISS-MODEL server. The GMQE value of the simulated protein was 0.01. The QMEANDisCo global score was 0.16 ± 0.12. (**a**) Estimated three-dimensional protein conformation in the ball-and-stick confirmation. (**b**) QMEAN Z-score analysis according to Benkert. (**c**) Estimated local quality of the simulated US11 protein.

Using the one-letter amino acid code, a possible three-dimensional structure of the recombinant US11 protein was obtained using the SWISS-Model server. The resulting three-dimensional protein conformation, shown in Figure 1a in the ball-and-stick confirmation, was simulated based solely on the amino acid sequence due to the lack of an available template in the SWISS-MODEL server database. The global model quality estimate (GMQE) value of the simulated protein was 0.01, indicating low expected quality, as this score ranges from 0 to 1. Similarly, the QMEANDisCo global score was 0.16 ± 0.12, further indicating the low quality of simulation. The QMEAN Z-score analysis, shown in Figure 1b, plots the protein size on the X-axis against the QMEAN score on the Y-axis. Black dots represent experimental structures with QMEAN scores within one standard deviation of the mean, while gray spots represent those within two standard deviations. Light gray dots indicate structures further from the mean, and the red star marks the simulated US11 protein. Additionally, the local quality of the simulated US11 protein, shown in Figure 1c, plots each residue on the X-axis against similarities to native structures on the Y-axis, with scores below 0.6 expected to be of low quality.

In summary, while the production and three-dimensional simulation of recombinant US11 proteins were successful, the protein simulation showed low-quality estimates. Nevertheless, these recombinant US11 proteins were subsequently used to stimulate keratinocytes at a final concentration of 0.1 mg per 1 ml of cell culture medium.

### 3.2. Stimulation of Primary Keratinocytes with Recombinant US11 Protein

**Figure 2:**
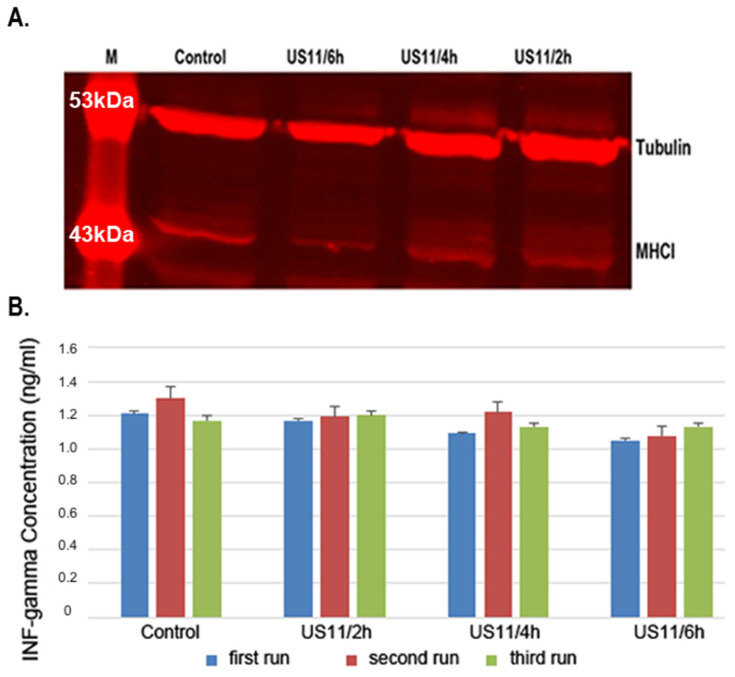
Western blotting and IFN-γ ELISA. (**A**) Western blotting results of keratinocytes stimulated with recombinant US11 proteins after 2, 4, and 6 h, indicating reduced MHC class I expression after 6 h compared to non-stimulated controls. The blot is cropped to the region of interest. (**B**) IFN-γ release of PBMCs was evaluated using IFN-γ ELISA. IFN-γ concentrations were measured 2, 4, and 6 h after US11 stimulation and compared to the corresponding controls. Three samples from each study group were randomly selected for measurement (first, second, and third run).

Keratinocytes were stimulated with the recombinant US11 protein for 2, 4, and 6 h, with non-stimulated keratinocytes as the controls. The Western blotting results are shown in Figure 2A. The red immunofluorescence is directly proportional to the measured protein concentration. In the 2-h samples, there were no differences compared to the non-stimulated controls (Figure 2A). A similar result was seen in the 4-h samples (Figure 2A). After a period of 6 h, a reduced MHC-I expression was observed, indicated by the decreased red fluorescence of the protein band compared to the non-stimulated controls (Figure 2A). Based on the semi-quantitative results of the Western blot, it can be stated that keratinocytes showed reduced expression levels of MHC-I after 6 h, whereas samples taken after 2 and 4 h showed comparable expression levels to the non-stimulated controls.

To assess immunogenicity, the co-cultivation of US11-stimulated keratinocytes with PBMCs was performed. Three samples from each study group were randomly selected for IFN-γ measurements (first, second, and third run). Each sample was measured in triplicate and the mean values with the corresponding standard deviations were calculated. The results are shown as bar graphs with the corresponding standard deviations in Figure 2B. After 2 h, the mean IFN-γ gamma concentrations of 1.17 ± 0.02 ng/mL, 1.19 ± 0.01 ng/mL, and 1.20 ± 0.01 ng/mL could be measured. After 4 h of stimulation, a decrease in the mean measured IFN-γ concentrations to 1.09 ± 0.05 ng/mL, 1.22 ± 0.03 ng/mL, and 1.13 ± 0.17 ng/mL could be observed. The final mean concentrations of IFN-γ were measured at 1.05 ± 0.10 ng/mL, 1.08 ± 0.10 ng/mL, and 1.13 ± 0.03 ng/mL 6 h post-stimulation. The control values of unstimulated keratinocytes were measured at 1.20 ± 0.01 ng/mL, 1.30 ± 0.02 ng/mL, and 1.17 ± 0.02 ng/mL. The IFN-γ ELISA measurements, shown in Figure 2B, revealed a trend toward decreasing IFN-γ concentrations over 2-, 4-, and 6-h periods, indicating a reduced immunological response; however, these results were not statistically significant.

**Figure 3:**
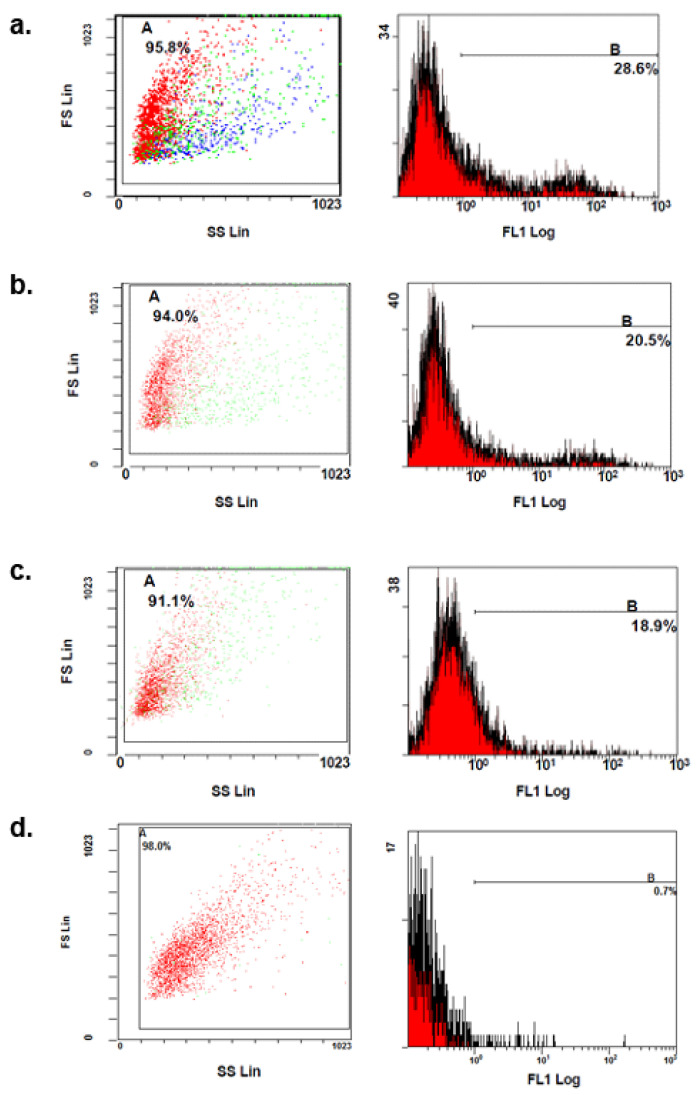
Flow cytometry. Each measuring point represents one measured cell. The X axis represents the relative fluorescence intensity of the dye, and the Y axis represents the number of cells. (**a**) Non-stimulated keratinocytes (controls) (**b**) 2 h post-stimulation; (**c**) 6 h post-stimulation. (**d**) Control measurements of IgG antibody isotype controls. (FS: forward scatter; SS: side scatter; lin: linear; log: logarithm; FL1: fluorescence of MHC-I.)

Flow cytometry further quantified MHC-I expression after 2 and 6 h of US11 protein stimulation. Because Western blot results at 4 h showed no differences compared with US11 stimulation at 2 h, flow cytometric measurement at 4 h was omitted and measurements were performed only at 2 and 6 h. Figure 3 shows histograms of MHC-I surface expression. The X-axis represents the relative fluorescence intensity of the dye, and the Y-axis represents the number of cells. A range was defined for which the measured intensities of the samples could be considered positive or negative. The marker line B in the right-handed graphs represents the positive range. The measured results were the following: 28.6% in non-stimulated controls (Figure 3a), 20.5% after 2 h (Figure 3b), and 18.9% after 6 h (Figure 3c). Control measurements of IgG antibody isotype controls were also performed (Figure 3d). When the sample values (Figure 3b,c) were plotted against the value of the controls, the relative antibody binding and down-regulation of MHC-I was obtained. The controls were defined as 100%. The cells examined 2 h after US11 stimulation showed a relative expression of 72.0% compared to the controls. This corresponds to a down-regulation of 28.0%. The sample examined 6 h after US11 stimulation showed an expression of MHC-I molecules of 66.0%. Compared to the controls, this corresponds to a relative down-regulation of 33.0%.

### 3.3. Treatment of Human Allogeneic Skin Tissue with US11 Protein

**Figure 4:**
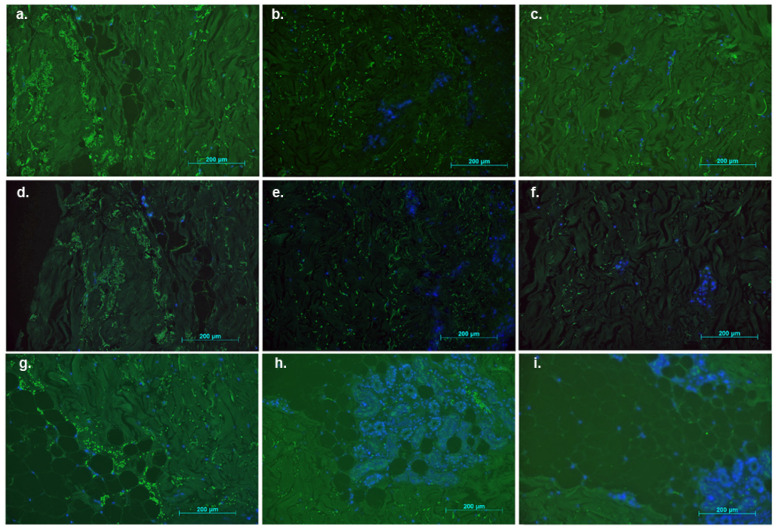
Immunofluorescence results of stimulated allogeneic skin samples. Human allogeneic skin samples were treated with the recombinant US11 proteins. The green fluorescent structures represent MHC-I molecules. The cell nuclei are shown as blue and fluorescent by DAPI. Every image represents a different sample. (**a**,**d**,**g**) Non-stimulated skin tissue after 7 days of culture (controls). (**b**,**e**,**h**) Stimulated skin tissue 7 days after stimulation with recombinant US11 proteins 6 times daily every 2 h. (**c**,**f**,**i**) Stimulated skin tissue 7 days after stimulation with recombinant US11 protein twice daily every 6 h.

Human allogeneic skin samples were treated with recombinant US11 protein several times daily over a 7-day period. The skin samples were treated with recombinant protein either every 2 or 6 h. The results are shown in Figure 4. The green fluorescent structures represent MHC-I molecules. The cell nuclei appear to be blue and fluorescent. Figure 4a,d,g shows the control measurements of three different non-stimulated skin samples after 7 days. The MHC-I expression is pronounced over a large area (Figure 4a,d,g). After stimulation of the samples with recombinant US11 protein six times daily every 2 h in a 12-h time window, a reduction in MHC-I expression was observed after 7 days (Figure 4b,e,h). Figure 4c,f,i shows immunofluorescence results after stimulation of three different skin samples with recombinant US11 protein twice daily every 6 h over a period of 7 days. A reduction in MHC-I expression could also be observed compared to controls. Overall, daily treatment of human skin samples with recombinant US11 protein appears to reduce MHC-I expression after a 7-day period, as indicated by reduced immunofluorescence. This reduction suggests effective down-regulation of MHC-I expression through US11 protein treatment, irrespective of the stimulation interval.

In conclusion, these findings highlight the potential of recombinant US11 protein to immunosilenced keratinocytes by reducing MHC-I expression and potentially create non-immunogenic skin substitutes for allotransplantation.

## 4. Discussion

To the author’s knowledge, this is the first study that investigated the successful immunomodulation of human primary keratinocytes using recombinant HHV US11 proteins in vitro. To date, the function as well as the impact of recombinant US11 protein in cell culture and animal models is poorly understood [50]. Based on the one-letter amino acid code, recombinant US11 protein was synthesized and became commercially available. In 2011, the expression and purification of herpes simplex virus type 1 US11 protein was first described [50]. It is known that the US11 protein is a 21 kDa, highly basic phosphoprotein functioning as an RNA-binding protein that post-transcriptionally regulates gene expression [51,52,53,54]. According to the manufacturer’s instructions, recombinant US11 protein used in the present study was synthesized using a bacterial system. Based on the one-letter amino acid code, a possible three-dimensional protein structure of the recombinant US11 protein was obtained using the SWISS-Model server. The resulting three-dimensional protein conformation showed low-quality estimates of the protein simulation as indicated by the low GMQE value and QMeanDisCo global score. Computer and server-based structural models are established as valuable complements to experimental studies, contributing between the known protein sequence and the experimentally determined tertiary or quaternary structure [49]. However, it is known that the computational simulation of protein structures based on amino acid codes remains a fundamental problem in biochemistry and is one of the most challenging issues in bioinformatics [55]. The SWISS-MODEL server provides an easily accessible and fully automated tool to create and visualize reliable protein structures [49]. Despite the rapid and ongoing developments of the SWISS-MODEL server, not all protein structures in nature can be captured yet, but the vast majority of sequences will be covered in the coming years [56,57]. Based on the fact that the recombinant protein used in the present study is of viral origin, there is a possibility that it is not yet recorded in any protein database. As this is the first presentation of the recombinant HHV US11 protein as a biopharmaceutical, there is no data in the literature for comparison regarding the simulation of its tertiary and quaternary protein structure. Due to limited financial resources, protein crystallization and corresponding investigation and imaging could not be performed yet. Therefore, protein crystallization of the recombinant US11 protein could be a valuable addition to future experiments and could add significantly to the published data regarding the tertiary and quaternary structure of the recombinant US11 protein. Due to the lack of protein crystallization data, no statement can be made at present about the correct protein folding of the recombinant US11 protein used. However, the corresponding functionality of the recombinant US11 protein used has been observed in all subsequent experiments, recognizable by the reduced MHC-I expression.

In a previous study by the authors, the effective transfection of human primary keratinocytes with adenoviral pCMV6-US11 vectors resulted in reduced MHC-I expression levels 24 h post-transfection, with levels returning to their previous expression levels after 48 h [15]. Over the past years, considerable progress has been made regarding the use of viral vectors for gene transfer into keratinocytes, making the application much safer and more precise [58,59]. Regarding the efficiency of MHC-I reduction, the results of the present study align with the previously published literature. In earlier studies, a relative decrease of 32.2% in MHC-I expression in human primary keratinocytes was observed 24 h post-transfection using an HCMV US11 vector, with re-increasing MHC-I expression levels after 48 h post-transfection, resulting in expression levels comparable to non-transfected controls [15]. In a study by Lee et al., human neuronal stem cells showed relatively decreased MHC- I expression levels, ranging from 20.0% to 50.0% compared to controls after transfection with US2, US3, US6, and US11 vectors [60]. Other studies showed similar results regarding decreased MHC-I expression, ranging between 25.0% and 96.0% using viral transfection [41,61]. However, there seems to be a cell type-specific variability in the reduction in MHC-I expression, which may limit immune evasive properties. As published earlier by Radosevich et al., different human cell lines showed significant variability in MHC-I reduction after transfection with US11 [61]. It remains uncertain to which level MHC- I expression has to be decreased in vivo to avoid host CD8+ T cell recognition or NK cell activation. Whereas the near-complete elimination of MHC-I on the cell surface is known to be a catalyst for NK cell-mediated apoptosis, it was reported that as few as 1 to 200 MHC- I molecules can induce a host immune cellular response [21,22,23,43].

The present study is the first of its kind to use recombinant HHV US11 protein as a biopharmaceutical to reduce MHC-I surface expression in human primary keratinocytes. Further extensive investigations are required to elucidate these findings and to place them in the broad context of the published literature. To circumvent the negative side effects of viral vectors, such as increased immunogenicity, induced oncogenesis, and restricted applications due to regulations, the presented experimental set-up abandoned the gene therapeutic approach and focused on the protein level to develop a novel biopharmaceutical to reduce the alloreactivity of keratinocytes [59]. According to current data, there are no studies that can be used as a comparison for the reduction in MHC-I surface expression in keratinocytes after successful stimulation using recombinant HHV US11 proteins. In the present study, Western blotting showed a semi-quantitative decrease in MHC-I expression 2 and 6 h post-stimulation with the US11 protein. The results were further quantified using flow cytometry. A reduction in MHC-I expression by 28.0% at 2 h and 33.0% at 6 h after stimulation with the recombinant US11 protein was detected. Therefore, the aim of MHC-I reduction on the cell surface of keratinocytes was achieved after 6 h. It was shown elsewhere that US2 and US11 transcripts appeared approximately 6 h after host infection with HCMV [62]. The findings presented within this study support the published literature based on CMV US11 vectors and proteins [15]. However, in the study at hand, MHC-I expression is not quantified over the period of 6 h. A rebound of MHC-I surface expression to the baseline levels could not be observed as published elsewhere [15]. Therefore, further studies beyond the 6-h period are needed to investigate the effect of US11 protein stimulation on keratinocytes.

The effects of recombinant HHV US11 proteins on an organism in vivo also remain unclear. To obtain a first impression of the immunogenicity of HHV US11-treated keratinocytes, co-cultivation with PBMCs and determination of the released IFN-γ by ELISA were performed in the study at hand. PBMC assays can be used to assess cell-mediated immunity and antigen-specific stimulation, and they can also be used to monitor the response to immunotherapies [63]. The IFN-γ release of PBMCs correlated with the immunogenicity of keratinocytes treated with the recombinant US11 protein. A moderate trend with reduced IFN-γ release over a period of 6 h was observed. However, further investigations, e.g., by enzyme-linked immune spot assay (ELISpot) or in vivo experiments, are required to further quantify the reduced immunogenicity [64]. As ELISpot assays are widely used methods for the determination of antigen-specific cytokine production or the activation of antibody-secreting immune cells, it could be valuable for future experiments [65]. The initial data presented in this study on the treatment of human skin with the recombinant HHV US11 protein for a period of 7 days showed a promising trend with reduced MHC-I expression in a complex tissue. It should also be noted that, according to current knowledge, it is unclear whether the cell properties of keratinocytes differ between post-bariatric patients and healthy donors after removal. However, there are no data in the literature to date that can be used for comparison. Therefore, further in vitro and in vivo studies are needed to quantify the potential clinical applicability, as well as the immune response of the recipient organism and allograft rejection.

If a reduction in MHC-I expression on primary allogeneic keratinocytes is not sufficient to prevent rejection after transplantation, different strategies could be considered to optimize the experimental approach in vitro. As the host immune cellular response is not only mediated by MHC-I-associated CD8+ cytotoxic T cell activation, multiple other parameters have to be taken into account. For example, multiple cytokines known to influence or even up-regulate the MHC-I presentation pathway have to be considered [66]. So far, most approaches modifying MHC-I expression using US proteins have been based on a transfer of corresponding genes, for example, using viral vectors [67]. It was observed that US2 and US11 utilize different targeting mechanisms to influence MHC-I expression [68]. Furthermore, it is of interest that data to date is mainly based on HCMV US11 proteins and further experiments are required using the HHV US11 protein of the present study [50]. An experimental approach that could combine the immunomodulatory abilities of different regulatory proteins would also be conceivable. For example, it was shown that the co-expression of US2 and US3 or US2 and US11 resulted in an almost complete reduction in MHC-I expression [68,69]. Complementary evidence was provided that US2 and US11 use similar mechanisms to degrade MHC-I molecules so that their effect could be summed and possibly potentiated based on previous findings [37,70,71]. However, a careful risk–benefit analysis should be performed because the use of multiple immunomodulatory factors could increase the immunogenicity of the modified cells. Another potential approach is the fast-growing field of immunoengineering to influence the reactivity of allotransplants and the recipient’s immune system. A micro- or nanoparticle-based drug delivery system that could bring recombinant HHV US11 proteins or RNA-based particles into the target cells could also be a promising approach to reduce alloreactivity of keratinocytes in vitro and in vivo [25,72]. However, there remain challenges in the potential immunoengineering applications of the US11 protein due to the manifold systems and players involved [73]. A systemic approach for the application of immunoengineering technologies has to be critically discussed, since MHC-I is expressed on every nucleated human cell and a systemic reaction should be avoided.

## 5. Conclusions

Overall, the present study showed a valuable proof of concept that recombinant US11 proteins could be utilized as biopharmaceutical to reduce the alloreactivity of keratinocytes and allogeneic human skin samples, but there are still further investigations needed. The study aimed to show that human primary keratinocytes can be immunomodulated by reducing MHC-I expression using recombinant US11 proteins in vitro. This novel biopharmaceutical could generate non-immunogenic keratinocytes, overcoming graft rejection in allotransplantation and avoiding the drawbacks of gene therapy. However, further data and research, particularly in vivo, are needed to better classify and deepen the findings of the present study. The long-term goal is to produce off-the-shelf transplantable allogeneic keratinocytes that mimic the biological function of original skin.

## Data Availability

The primary data that support the findings of this study are available from the corresponding author upon reasonable request.

## Abbreviations

The following abbreviations are used in this manuscript:

β2m	β2-microglobulin
CD	Cluster of differentiation
DAPI	4′,6-Diamidino-2-phenylindol
ELISA	Enzyme-linked immunosorbent assay
ELiSpot	Enzyme-linked immunosorbent spot
GMQE	Global model quality estimate
HCMV	Human cytomegalovirus
HHV	Human herpes virus
HLA	Human leukocyte antigen
IgG	Immunoglobulin G
IFN-γ	Interferon gamma
MHC-I	Major histocompatibility complex class I
NK	Natural killer cells
PBMC	Peripheral blood mononuclear cells
PBS	Phosphate-buffered saline
PVDF	Polyvinylidene fluoride membrane
RNA	Ribonucleic acid
SDS-PAGE	Sodium dodecyl sulfate-polyacrylamide gel electrophoresis
US	Unique short glycoprotein
QMEAN	Qualitative model energy analysis
